# Prenatal Diagnosis of Treacher-Collins Syndrome Using Three-Dimensional Ultrasonography and Differential Diagnosis with Other Acrofacial Dysostosis Syndromes

**DOI:** 10.1155/2013/203976

**Published:** 2013-04-04

**Authors:** Daniela Cardoso Pereira, Luiz Claudio Silva Bussamra, Edward Araujo Júnior, Carolina Leite Drummond, Luciano Marcondes Machado Nardozza, Antonio Fernandes Moron, José Mendes Aldrighi

**Affiliations:** ^1^Department of Obstetrics and Gynecology, School of Medical Sciences, Santa Casa de São Paulo (FCMSCSP), São Paulo, SP, Brazil; ^2^Department of Obstetrics, Federal University of São Paulo (UNIFESP), Rua Carlos Weber 956, Apartamento, 113 Visage, Vila Leopoldina, 05303-000 São Paulo, SP, Brazil

## Abstract

Treacher-Collins syndrome (TCS) is a rare dominant autosomal anomaly resulting from malformation or disruption of the development of the first and second branchial arches. It is characterized by micrognathia, malar hypoplasia, and malformations of the eyes and ears. The prenatal diagnosis using two-dimensional ultrasonography (2DUS) is characterized by identification of facial malformations together with polyhydramnios. Three-dimensional ultrasonography (3DUS) has the capacity to spatially display these facial malformations, thus making it easy for the parents to understand them. We present a case of TCS diagnosed in the 33rd week using 3DUS, with postnatal confirmation using cranial computed tomography and anatomopathological analysis.

## 1. Introduction

Treacher-Collins syndrome (TCS) is a rare dominant autosomal craniofacial disorder (1 : 50,000) characterized by symmetrical bilateral malformations that frequently include hypoplasia of the mandibular-zygomatic complex, palpebral fissures, coloboma of the lower eyelid, and absence of medial eyelashes at the defect, malformation of the middle and external ears, and conductive hearing loss [1]. More than 60% of the cases do not present any family history and are new mutations. The gene penetrance of TCS seems to be high, but the severity of the malformations varies very widely within and between families [2]. TCS forms part of the craniofacial dysostosis group, along with the Goldenhar and Nager syndromes [3].

Diagnosing TCS prenatally is very important because the tongue may obstruct the airways and lead to the newborn's death. A prenatal diagnosis of this syndrome may be suspected from two-dimensional ultrasonography (2DUS), through identifying the presence of polyhydramnios, micrognathia, palpebral clefts, and low-set ears [3, 4]. Three-dimensional ultrasonography (3DUS) has been used for diagnosing certain fetal abnormalities [5, 6], given that in rendered mode it allows the parents to understand the malformations better. This facilitates termination in unviable cases, in countries where the law permits this. In relation to TCS, there are only two reports in the literature, both published 10 years ago [7, 8].

We present a case of TCS diagnosed in the 33rd week by means of 3DUS, with postnatal confirmation of the anomalies by means of computed tomography (CT) on the face. We emphasize the differential diagnoses with other types of craniofacial dysostosis, along with the importance of 3DUS for enabling the parents to have a better understanding of the facial malformations.

## 2. Case Report

**Figure 1:**
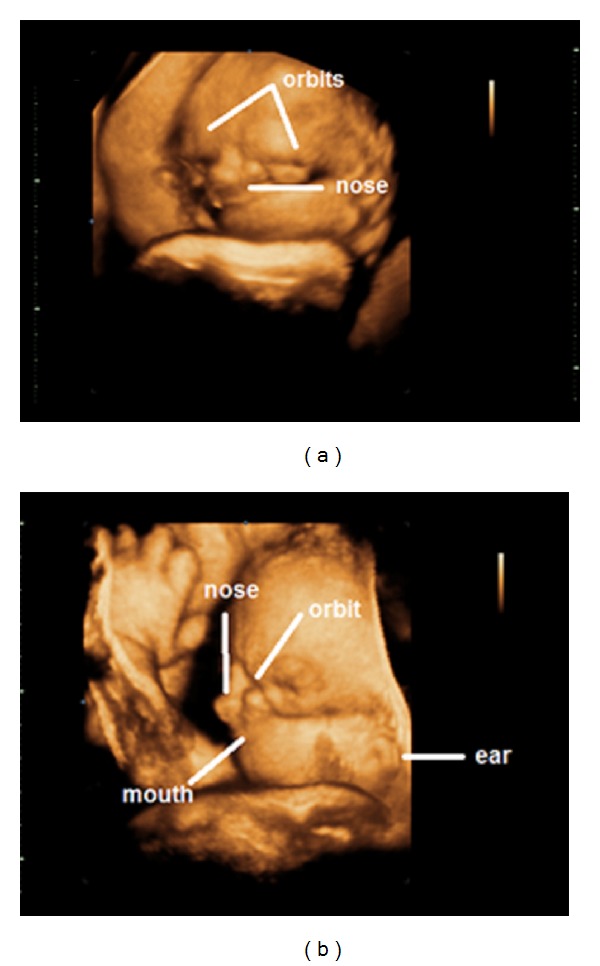
(a) and (b) Three-dimensional rendering image of the fetal face, showing transverse facial fissure, microphthalmia, and low-set ears.

The patient was a 24-year-old primigravida without comorbidities and with adequate levels of prenatal care. She was referred to the Department of Gynecology and Obstetrics, School of Medical Sciences, Santa Casa de Misericórdia de São Paulo (FCMSCSP), because of a suspicion of severe facial malformation in the fetus, seen on obstetric level I ultrasonography at another clinic. A 2DUS examination performed in the 33rd week showed microphthalmia, a transverse facial cleft occupying the lower half of the face and bilateral auricular hypoplasia. A “golf ball” was also viewed in evaluating the four heart chambers, which was confirmed by means of fetal echocardiography. However, there were no other associated cardiac malformations. In order to view the face of the fetus better, 3DUS was performed using the SonoAce X8 device (Samsung-Medison, Seoul, Republic of Korea), equipped with a volumetric convex transducer (3–7 MHz). The 3D image in rendering mode made it possible to clearly view the transverse facial cleft, microphthalmia, and low-set ears (Figure 1). In the light of the severe facial malformations, in the absence of other associated malformations, the hypothesis of TCS was raised. The 3D image was fundamental for allowing the parents to have a better understanding of the poor postnatal prognosis.

**Figure 2:**
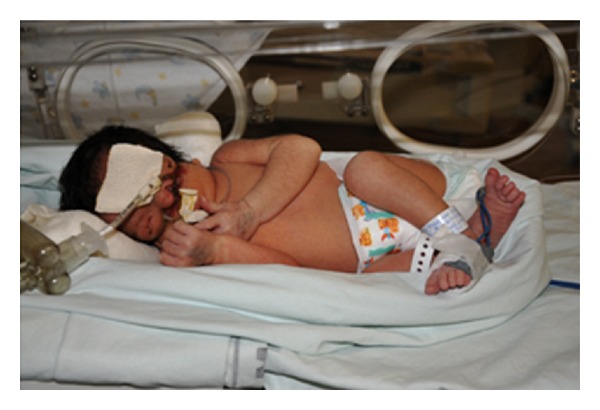
Image of the newborn showing micrognathia, maxillary hypoplasia, anophthalmia, bilateral auricular appendages, and severe microtia.

**Figure 3:**
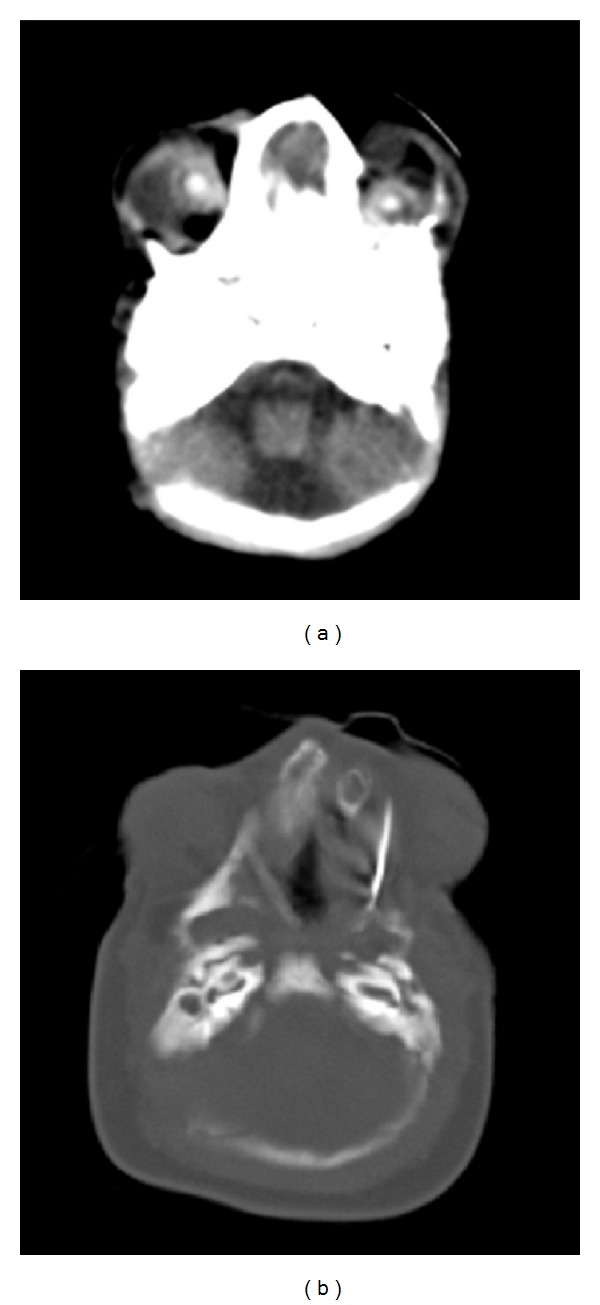
Computed tomography of the cranium, in the axial plane, showing severe hypoplasia of the maxillary bones (a), which were dysmorphic and covered with skin (b).

The patient underwent cesarean delivery at a gestational age of 35 weeks and 3 days because of fetal hemodynamic centralization, giving birth to a live female infant weighing 2,355 g and presenting Apgar 7 and 8. Due to respiratory discomfort, the newborn was provided with orotracheal intubation early on. The physical examination conducted after the birth showed micrognathia, hypoplasia of the maxilla, anophthalmia, bilateral auricular appendages, and severe microtia (Figure 2). Cranial CT confirmed the findings of the physical examination and also showed the presence of severe hypoplasia of the maxillary bones. These were dysmorphic and covered with skin, and an extensive infraorbital side-to-side cleft was identified (Figure 3). The limbs, spine, and other organs did not present any abnormalities on clinical and radiological assessment. 

Extubation was not possible because of the severe facial deformity, which affected the respiratory canals, and therefore, the newborn was subjected to tracheostomy and gastrostomy. Eleven days after birth, the newborn progressed to death due to bronchopneumonia. The autopsy confirmed the physical and imaging findings.

## 3. Discussion

Facial dysmorphism, particularly when associated with disorders of the first and second branchial arches, generally results from a combination of migration and inadequate formation of facial mesenchyme [9]. Several syndromes have been described, among which the main ones are Pierre Robin, hemifacial microsomia, Goldenhar, Treacher-Collins, Nager, and velocardiofacial syndromes. The main classifications of craniofacial malformations were provided by Tessier [10], who correlated the anatomical site of the abnormality with the medial line axis, and by van der Meulen et al. [11], who correlated malformations at the time of embryo development and divided them into internasal, nasal, nasal-maxillary, and maxillary. The main differential diagnoses of TCS are with the Goldenhar and Nager syndromes. Goldenhar syndrome is characterized by ear malformations, hemifacial microsomia, a lateral facial cleft, ocular abnormalities, and vertebral abnormalities [12]. Nager syndrome is characterized by ear abnormalities, micrognathia, radial hypoplasia of the limbs, and absence of thumbs and/or other fingers [13]. In our specific case, the diagnosis of TCS was defined by the presence of facial malformations in the absence of abnormalities in other segments.

In our case, the diagnosis of TCS was made in the 33rd week of pregnancy. Late diagnosis is generally due to late-starting prenatal followup at public healthcare services in developing countries like Brazil. Moreover, since Brazilian law does not permit termination of pregnancy in cases of fetal malformation, this contributes towards late diagnosis of severe fetal malformations, thus putting the pregnant women's lives at risk. Prenatally, TCS diagnosed by means of 2DUS is well established through findings of severe facial abnormalities, generally in association with polyhydramnios, which is an indirect sign of upper airway obstruction. Therefore, adequate prenatal diagnosis enables followup for the pregnant woman at a tertiary-level service, so as to provide better support for the newborn. 

With regard to 3DUS, there are only two reports in the literature of prenatal diagnosing of TCS, and both of these cases were also diagnosed at a late stage [7, 8]. Both of the previous cases were described 10 years ago, at a time when the resolution of the rendering 3D images (Voluson 530, Kretztechnik, Zipf, Austria) was still relatively poor. Nonetheless, in both of those cases, 3DUS clearly showed the facial malformations typical of TCS. On the other hand, there were no descriptions of use of 3DUS to allow the parents to understand the situation better. In comparison with 2DUS, 3D images have been proven to increase the maternal-fetal interaction [14]. This greater interaction may facilitate the decision to terminate the pregnancy in cases of severe fetal malformation. In our specific case, the 3D image was of fundamental importance in helping the parents to better recognize the severe facial malformation. Nonetheless, because of the advanced gestational age, it was decided to continue with followup of the gestation, although this was subsequently terminated through cesarean delivery because of fetal distress. 

In summary, we presented here a case of TCS diagnosed in the third trimester by means of 3DUS, which was confirmed postnatally by means of CT on the face and anatomopathological analysis. We believe that 3DUS should be used in cases of severe fetal abnormalities, so that the parents can understand the malformations better.
